# Factors associated with under-five child mortality: an analysis of the Rwanda Demographic Health Survey (RDHS) 2019/2020

**DOI:** 10.11604/pamj.2025.51.105.44386

**Published:** 2025-08-26

**Authors:** Julienne Nyirarukundo, Boniface Nsengiyumva, Rosemary Okova, Ancille Murekatete

**Affiliations:** 1 Department of Public Health, Mount Kigali University, Kigali, Rwanda,; 2 Department of Public Health, University of Rwanda, Kigali, Rwanda

**Keywords:** Child mortality, health survey, Rwanda

## Abstract

**Introduction:**

under-five mortalities (U5M) are significant in determining the level of health development of a country. Most countries work to reduce the U5M rate; however, not all achieve the goal, including Rwanda. This study intends to explore factors associated with under-five mortality in Rwanda.

**Methods:**

the study is a quantitative retrospective study using cross-sectional survey data of the Rwanda Demographic Health Survey 2019-2020. Permission for access to data was obtained from the DHS program website. The children dataset (KR) was used with a total of 8092 children. Data analysis used STATA-15 software. Descriptive information had been presented using frequency tables, charts, and graphs. A bivariate analysis using Chi-square was conducted to determine factors associated with U5M. Variables with significant association (P<0.05) in the bivariate analysis were considered for multivariate analysis with logistic regression. P-value <0.05 was declared as a significant association.

**Results:**

the mortality level in 2019/2020 was at 3.66% linked to maternal and child-related factors; low birth weight was (AOR=6.93, 95% CI=4.44-10.81). Postnatal checkup was (AOR= 0.42, CI=0.22-0.81); (P-value <0.009). Delivery at health facility (AOR =0.32, CI=0.13-0.77); (P-value =0.011). The significant socio-economic factors of U5M were the number of household members (p-value=0.002), (AOR=0.59, 95% CI=0.42-0.83) and the partner´s education of secondary level (OR=0.47,95% CI=0.27-0.82); (P-value=0.008) and higher level (AOR=0.29,95% CI=0.11-0.78); (P-value=0.015).

**Conclusion:**

results showed that low birth weight and inadequate post-natal check-up visits are the main associated factors of under-five child mortality. In contrast, the remaining factors were all found to be protective.

## Introduction

Under-five child mortality, also known as under-five mortality (U5M) or child mortality, refers to the number of deaths of children under the age of five per 1000 live births. It is one of the sensitive indicators used to assess children and overall population well-being, especially in terms of nutrition, healthcare, and socio-economic status [1].

Globally, the under-five mortality level in 1990 was at 93 deaths per 1,000 live births, the highest rate, which fortunately decreased progressively by 60% to reach 37.7 deaths per 1,000 births around 2019 and 2020 [2].

The highest mortality rate was experienced by sub-Saharan Africa (SSA) alone, which carried half of the burden with approximately 2.8 million deaths. Central and Southern Asia had 1.5 million (28%), while regions like Australia, New Zealand, Eastern and South-Eastern Asia, Northern Africa and Western Asia, Europe and Northern America, Latin America, Caribbean and Oceania are carrying 19% of under-five deaths [3].

In Eastern African countries, the level of U5M was 51.31 per thousand live births. According to studies conducted among 12 countries from 2008 to 2019. Rwanda was among them and was the sixth on the list with a pooled incident rate of 38.61 (38.61, 38.61), 7.55% Kenya was the first East African country with a high mortality rate, 16.29%, followed by Malawi, 13.17% Uganda, 11.06%1, Madagascar, and Burundi, around 8% and so on. Most causes linked to U5M are all preventable [4]. The highest mortality rate was experienced in sub-Saharan Africa (SSA), which alone carried half of the burden with approximately 2.8 million deaths. Central and Southern Asia had 1.5 million (28%), while the regions like Australia, New Zealand, Eastern and South-Eastern Asia, Northern Africa and Western Asia, Europe and Northern America, Latin America, Caribbean, and Oceania are carrying 19% of under-five deaths [4].

In Rwanda, the progressive U5MR reduced from 196 in 2000 to 45 deaths per 1000 live births in 2019-2020. The highest rate of those deaths was mainly observed in 3 provinces of the country, whereby 56-57% was in East, North, and South, and the lowest was in West, 44% and in Kigali city, 31%. Mother´s education, household wealth index, and poverty have been linked with U5M in that period [5].

In order to achieve that progress, the greatest effort has been made by Rwanda through a community-based approach to improve maternal and child health [6]. Despite major interventions made throughout the country to increase child survival, some communities still experience child death, mainly in rural areas rather than urban areas. This study intends to look for factors associated with under-five mortality in Rwanda in order to inform policymakers and significant others to emphasize their efforts and support to eradicate under-five child mortality.

## Methods

**Study design:** a quantitative retrospective study using cross-sectional survey data on Rwandan children gathered during the RDHS 2019-2020 was conducted to determine factors associated with under-five child mortality.

**Study setting and population:** the study was conducted in Rwanda, a landlocked country in Central/Eastern Africa, bordered by the Democratic Republic of the Congo, Uganda, Tanzania, and Burundi. Rwanda, with a population of 13,954,471, is divided into four provinces and the city of Kigali, further subdivided into 30 districts, 416 sectors, 2,148 cells, and 14,837 villages. The study focused on children under five years of age, using data from the Rwanda Demographic Health Survey conducted between November 2019 and June 2020. The target population comprised 8,092 children, with a nearly equal gender distribution: 50.52% male and 49.48% female, based on the 2012 Rwanda Population and Housing Census.

**Participants:** the study involved 8,092 children under the age of five, selected from the 2019/2020 Rwanda Demographic Health Survey (RDHS), which covered 13,000 households. Eligibility criteria included all children under five years old from the selected households, with a response rate of 99.7%. The RDHS employed a two-stage sampling technique: first, 500 enumeration area (EA) clusters (388 rural, 112 urban) were selected from the 2012 Rwanda Population and Housing Census (RPHC). In the second stage, a systematic sample of households was randomly chosen within each EA cluster. All eligible children in these households were included in the study. Data was obtained from the DHS program website.

**Variables:** the variables of the study included under-five child mortality as the primary outcome, defined as the death of children under the age of five. Exposure variables comprised low birth weight (less than 2,500 grams), the number of antenatal care (ANC) visits during pregnancy (1-3 vs 4 or more), postnatal checkup, place of delivery (health facility vs elsewhere), malaria prevention (use of mosquito nets), and anemia status (based on hemoglobin levels). Predictor variables included maternal age, maternal education level, maternal occupation, partner´s education level, partner´s occupation, and household size. Potential confounders were household head gender, residence (urban vs rural), religion, and marital status. Effect modifiers included birth order (first to third born) and birth interval (less than 2 years vs 2 years and above). Diagnostic criteria involved anemia, diagnosed by hemoglobin levels, and low birth weight, defined as birth weight under 2,500 grams.

**Data resource and measurement**

**Data collection tool:** the study utilized data from the RDHS 2019/2020, which included five questionnaires. Key tools were the household, men´s, and women´s questionnaires, gathering information on children´s health, maternal and child characteristics, and related factors. The women´s questionnaire focused on women aged 15-49, covering a wide range of health and demographic topics [5].

**Data collection:** RDHS 2019/2020 data sets were used after the researcher obtained approval from the required institutions: Mount Kenya University´s clearance and the Rwanda Demographic Health Survey. The RDHS children dataset contains answers for questions related to children, but given by the mothers. Furthermore, this dataset contains certain variables from households, women, and men that are somehow related to the children [5]. The RDHS data were kept anonymous, and participant information was kept private and safe by using a password-protected computer to store all the data and analysis outputs. They were not used for anything else except the aim of this study.

**Sample size:** the sample size for this study, derived from the 2019/2020 Rwanda Demographic and Health Survey (RDHS), includes 8,092 children under the age of five. This was part of a broader survey involving 13,000 households and 14,675 women aged 15 to 49, with a response rate of 99.7%. Advanced sampling techniques were used, including the Taylor linearization method for estimating parameters like averages and proportions, and jackknife replication for variance estimation. The probability of selecting each enumeration area (EA) was carefully calculated using specific population data, ensuring accurate representation. All children under five in selected households were included. Sampling errors were computed using SAS programs, ensuring the reliability of the study's findings.

**Data analysis:** all of the analyses were completed using STATA15. Descriptive analysis was used to analyze the demographic traits of the respondents. Frequency tables, charts, and graphs were used for the presentation of findings. Bivariate analysis was used to examine the relationship between respondents' sociodemographic characteristics and dependent variables. At this stage, the Chi-square test was used for qualitative variables to identify whether they are associated with under-five mortality. Multivariate logistic regression was used to adjust for confounding factors to determine the relationship of independent variables with its outcome. The corresponding adjusted odds ratios (AOR), 95% confidence intervals (CI), and p-values (<0.05) were considered to assess the statistical significance of the association. All analyses were weighted on primary sampling units and strata from rural and urban areas within each district of Rwanda.

**Ethical considerations:** this study adhered to strict ethical considerations. Approval was granted by Mount Kenya University, Rwanda, enabling access to data from the DHS program under ICF, with an official approval letter provided. Data confidentiality was maintained rigorously; the RDHS datasets did not include respondent names, ensuring anonymity. Participant information was securely stored on a password-protected computer, with access restricted to authorized personnel only. The data were used solely for the purposes outlined in the study´s objectives, ensuring that no personal information was exploited beyond the scope of this research. These measures were implemented to protect participant privacy and uphold ethical research standards.

## Results

**Sociodemographic characteristics:** many of the children were male, 50.52% (4,205 children), and the majority were aged between 24 to 59 months, 59.53% (5,955 children). Ninety-three and eight hundredths percent (93.08%) of the children had normal birth weight, while 6.92% had low birth weight. The prevalent birth order was first to third, encompassing 65.92% of the cases. The majority of children had a birth interval of 2 years and above, constituting 85.17%. Antenatal care (ANC) visits of many children were reported to be between 1 and 3 for 50.52%, while the majority (81.94%) did not undergo postnatal checkups. In terms of anemia, 87.49% of the children were found not to be anemic as per Table 1.

**Table 1:** characteristics of study participants (N=8325)

Variable	Frequency	Percentage
**Sex of child**		
Male	4205	50.52
Female	4119	49.48
**Age of child (months)**		
1-11 months	1675	20.12
12-23 months	1694	20.35
24-59 months	4955	59.53
**Birth weight**		
Normal	7333	93.08
Low	545	6.92
**ANC visits**		
No visit	143	2.276
1-3 visits	3184	50.52
4 visits	2975	47.2
**Postnatal checkup within 2 days**		
No	5164	81.94
Yes	1138	18.06
**Place of delivery**		
Home	445	5.35
Health facility	7754	93.15
Other	125	1.5
**Partner's education**		
No education	916	13.21
Primary	4654	67.13
Secondary	961	13.87
Higher	383	5.52
don't know	19	0.27
**Number of HH members**		
1-5	4988	53.93
6-10	3256	39.11
11 and above	80	0.96

ANC: antenatal clinic; HH: household

In addition, 93.15% of the children were born at a health facility. Concerning malaria prevention, the majority of children (53.07%) slept under a mosquito net. Regarding maternal characteristics, most mothers fell within the age range of 25 to 34 years (49.31%) and had attained a primary education level (64.88%). The prevalent occupation for mothers was farming (42.8%). Similarly, the majority of mothers' partners had a manual occupation (40.53%) and a primary level of education (67.13%) in Table 1.

Household characteristics indicated that 77.32% of the household heads were predominantly male, while 22.68% were female. The residence of a significant proportion of study participants was in rural areas (82.53%). The majority of households were identified as Protestants (50.41%). Marital status was predominantly married (50.46%). Most households had 1 to 5 members, accounting for 53.93%. The majority of children under five years old were between one and two years old (90.35%). Concerning access to healthcare, 76.23% of respondents did not find the distance to the health facility from their households to be a significant problem. Furthermore, the drinking water source for the majority of children was improved (43.58%), while the prevailing sanitation method was the use of pit latrines (94.2%) in Table 1.

The prevalence of under-five mortality among children aged under five years who were selected for this study was 3.66% (296 children). The children who were alive during RDHS 2019/2020 were 96.34% (7,796 children). When linking sociodemographic characteristics to analyze maternal and child-related factors of under-five child mortality, the analysis demonstrated only four factors to be in association with the low birth weight (p-value<0.001), the antenatal visits (p-value<0.001), the postnatal checkup (p-value=0.014) and the place of delivery (p-value=0.009) in Table 2.

**Table 2:** bivariate analysis results of maternal and child-related factors associated with under-five mortality in Rwanda using the Rwanda Demographic Health Survey 2019/2020 (N=8325)

Variable	Not died (%)	Died (%)	P-value
**Birth weight**			**P<0.001**
Normal	7142(97.39)	191(2.61)	
Low	485(88.93)	60(11.07)	
**ANC visits**			**<0.001**
No visit	134 (93.41)	10(6.59)	
1-3 visits	3095(97.22)	89(2.781)	
4 visits	2928(98.45)	46(1.55)	
**Postnatal checkup within 2 days**			**0.014**
No	5034(97.48)	130(2.52)	
Yes	1124(98.75)	14(1.25)	
**Anemia level**			**0.663**
Severe	9(100)	0(0)	
Moderate	131(94.34)	8(5.66)	
Mild	363(95.85)	16(4.15)	
Not anemic	3545(96.4)	132(3.60)	
**Place of delivery**			**0.009**
Home	405(90.87)	40(9.13)	
Health facility	7497(96.69)	257(3.31)	
Other	118(94.41)	7(5.59)	
**Children under 5 sleeping in a mosquito net**			**0.166**
No	862(97.28)	20(7.73)	
All children	4187(97.65)	101(2.35)	
Some children	704(98.95)	8(1.05)	
No net in HH	2139(97.5)	55(2.50)	
**Distance to health facility**			**0.947**
A big problem	1907(96.37)	71.84(3.63)	
Not a big problem	6112(96.33)	232.7(3.67)	

ANC: antenatal clinic; HH: household

**Maternal and child-related factors:** maternal and child-related factors; low birth weight was (AOR=6.93, 95% CI=4.44-10.81). Postnatal checkup was (AOR=0.42, CI=0.22-0.81); (p-value <0.009). Delivery at a health facility (AOR =0.32, CI=0.13-0.77); (p-value =0.011) in Table 3.

**Table 3:** multivariate analysis of maternal and child-related factors associated with under-five child mortality (N=8325)

Variable	Adjusted odds ratio	P-value	95% CI	
**Birthweight**				
Normal	1.00			
Low	6.93	<0.001	4.44	10.81
**ANC visits**				
0-2 visits	1.00			
1-3 visits	0.85	0.773	0.27	2.63
visits and above	0.58	0.368	0.17	1.92
**Postnatal check-up**				
No	1.00			
Yes	0.42	0.009	0.22	0.81
**Place of birth**				
Home	1.00			
Health facility	0.32	0.011	0.13	0.77
Other	0.40	0.425	0.04	3.75
Constant	0.07	<0.001	0.02	0.22

ANC: antenatal clinic

The bivariate analysis of sociodemographic factors of under-five child mortality revealed that only partner´s education (p-value=0.014) and household members (p-value < 0.05) were associated with under-five child mortality in Table 4 and Table 4.1.

**Table 4:** bivariate analysis of socio-economic factors associated with under-five mortality in Rwanda using the Rwanda Demographic Health Survey 2019/2020 (N=8325)

Variable	Not died (%)	Died (%)	P-value
**Sex of child**			**0.536**
Male	4046(96.2)	160(3.8)	
Female	3974(96.48)	145(3.52)	
**Age of child (months)**			**0.158**
1-11 months	1627(97.14)	8(2.86)	
12-23 months	1633(96.38)	61(3.62)	
24-59 months	4760(96.06)	196(3.94)	
**Birth order**			**0.157**
1-3 order	5302(96.62)	186(3.38)	
4-8 order	2594(95.91)	111(4.09)	
9-13 order	123(93.6)	9(6.40)	
**Birth interval**			**0.177**
Below 2 years	878(95.44)	187(3.54)	
2 years or above	5098(96.46)	187(3.54)	
**Mother's age**			**0.193**
15-24 years	1214(96.36)	46(3.65)	
25-34 years	3723(97.23)	106(3.51)	
35-49 years	2582(96.49)	94(3.17)	
**Mother's education**			**0.115**
No education	914(95.46)	43(4.53)	
Primary	5197(96.22)	204(3.78)	
Secondary	1555(96.76)	52(3.24)	
Higher	354(96.76)	5(1.44)	
**Mother's occupation**			**0.074**
No occupation	1425(97.48)	37(2.52)	
Farming	3423(96.08)	140(3.92)	
Services	985(95.35)	48(4.65)	
Manual	2083(96.29)	80(3.71)	
Domestic	104(100)	0	
**Partner's occupation**			**0.571**
No occupation	461(95.73)	20.53(4.27)	
Farming	2613(96.22)	102.5(3.78)	
**Partner's education**			**0.014**
No education	879(95.94)	37.2(4.06)	
Primary	4477(96.22)	176.1(3.78)	
Secondary	941(97.85)	20.72(2.16)	
Higher	378(98.4)	4.824(1.26)	
Don't know	17(94.83)	1(5.17)	
**Sex of HH head**			**0.234**
Male	6211(96.49)	226(3.51)	
Female	1809(95.82)	79(4.18)	

HH: household

**Table 4.1:** bivariate analysis of socio-economic factors associated with under-five mortality in Rwanda using the Rwanda Demographic Health Survey 2019/2020 (N=8325)

Variable	Not died (%)	Died (%)	P-value
**Place of residence**			**0.182**
Urban	1411(97.08)	42(2.92)	
Rural	6608(96.19)	262(3.81)	
**Religion**			**0.914**
No religion	75(97.71)	2(2.29)	
Catholic	2663(96.49)	97(3.41)	
Protestant	4042(96.34)	154(3.66)	
Tradition	1(100)	0	
**Marital status**			**0.091**
Never in	684(95.9)	30 (4.1)	
Married	4069(96.88)	131(3.12)	
Living with	2623(96.03)	108(3.97)	
Widowed	105(94.32)	6(5.68)	
Divorced	170(93.01)	13(6.99)	
Separated	368(95.72)	304(3.66)	
**Number of HH members**			**0.002**
1-5 members	4771(95.65)	217(4.35)	
10 members	3170(97.38)	85(2.62)	
11 members and above	78(97.03)	2(2.971)	
**Number of children under 5 in HH**			**0.057**
1-2	7224(96.06)	296(3.94)	
3-4	780(98.97)	8(1.03)	
5-7	15(100)	0	
**Drinking water source**			**0.533**
Improved	3504(96.58)	124(3.42)	
Unimproved	428(96.65)	15(3.35)	
Other source	4088(96.1)	166(3.9)	
**Sanitation facility**			**0.312**
Improved	356(97.72)	8328(2.28)	
Pit latrine	7551(96.3)	290(3.70)	
No toilet	8020(96.34)	5897(3.66)	

HH: household

**The significant socio-economic factors:** these were the number of household members (p-value=0.002), (AOR=0.59, 95% CI=0.42-0.83) and the partner´s education of secondary level (OR=0.47, 95% CI=0.27-0.82); (p-value=0.008) and higher level (AOR=0.29, 95% CI=0.11-0.78); (p-value=0.015) in Table 5.

**Table 5:** multivariable analysis of socio-economic factors associated with under-five child mortality in Rwanda, Rwanda Demographic Health Survey 2019/2020 (N=8325)

Variable	Odds ratio	P-value	95% CI	
**HH members**				
1-5 members	1.00			
6-10 members	0.59	0.002	0.42	0.83
**Partner's education**				
No formal education	1.00			
Primary	0.91	0.607	0.62	1.32
Secondary	0.47	0.008	0.27	0.82
Higher	0.29	0.015	0.11	0.78
Don't know	1.23	0.834	0.18	8.64
Constant	0.05	<0.001	0.04	0.07

HH: household

## Discussion

The analysis of study findings revealed a range of factors associated with under-five child mortality. The main and major cause of child mortality among all other factors revealed in this study was the low birth weight (11.07%) and post-natal visits (2.25%). This was the first study that discover findings in Rwanda. The remaining factors revealed were all found to be protective of child mortality. This is obvious and understandable because a lot of work has been done to reduce U5M. Other similar studies conducted by countries abroad were examined and discussed, comparing them with my study findings.

**Maternal and child-related factors of U5M:** the first objective of this study was to identify maternal and child-related factors. Concerning maternal and child-related factors, which were significant, low birth weight, post-natal care, and place of delivery are the main factors associated with U5M. This study found that in Rwanda, only 1138 (18%) of women have had their post-natal care within 2 days following the birth of the baby. This problem has a negative impact on the mother´s life and childhood outcome, because all post-partum complications, such as hemorrhage, retained membranes, and other child-related birth complications, can be discovered, reported, and managed on time. There are six aspects to postpartum care, including the postpartum check-up, medical attention, follow-up, health education, family planning services, and a healthcare system aiming to lower child morbidity and mortality. The guidelines state that a positive postnatal experience is defined as one in which women, children, partners, parents, caregivers, and families receive information, reassurance, and support in a consistent manner from motivated health workers [7].

The issue of not attending post-natal checkups is common in many countries in the sub-Saharan region, where 43% of women attended postnatal visits within the first 2 days of labor [8]. The postnatal during that period was low with 17% in Ethiopia, 43% in Malawi, and 49% in Guinea. The reason for not doing post-natal check-ups was linked with a lack of information about post-natal care and its importance to the health of the mother and that of the child. The post-natal check-up was still low when compared with Rwanda, with coverage in other Eastern African countries, where the overall result was 31.7%, as per studies conducted in 2020. The main reason for that is associated with the maternal education status, age of mother, community awareness, and healthcare worker provider characteristics [9].

Post-natal check-up attendance in the Northwest of Ethiopia was very high in 2018, with 57.5% (more than half), and it was about three times the one found in Rwanda. This big difference may be explained by various reasons, such as cultural differences, family support, and health care service provision, which is very widespread in that region [9].

Another factor associated with U5M was the place of delivery, which was much appreciated in Rwanda, with most women having a birth at health facilities and being attended by skilled health workers in public health facilities. Giving birth at a health facility has a positive outcome of labor since most complications that would occur are prevented. This result is valued compared to many other countries in the region and globally (66%). The result is greater than one found in Chad (23%) and is slightly lower than one found in Gabon (94%) [10].

The findings are higher compared with Bangladesh (40%), Nepal (62%), and Pakistan (69%). This inequality within countries was related to various factors in each country, and some of them are mother and partner´s education, watching television (in Bangladesh), and occupation [11]. A study done in 2018 about health facility delivery in sub-Saharan Africa revealed that women with at least primary education were twice as likely to give birth in facilities as women with no formal education [10,11].

**Child-related factors:** about child-related factors, low birth weight was the only factor associated with under-five mortality, and children who died because of low birth weight were 545 (11, 07%). This result is not high when it is compared with low birth weight (LBW) results of 15-20%, 28% occurring in the South Asia and sub-Saharan Africa (SSA) region in general, which had 13% of LBW. However, it is high when compared with the East Asian region at 6% and the Pacific and Latin American regions at 9%. The reason for these variations is that Rwanda is one of low-income countries where studies have shown that half of LBW children occur and mainly in susceptible populations. Again, that difference may be explained by the population size and economic state, coupled with advanced technology that makes life better in those countries [12].

Complications associated with low birth weight are many and have a negative impact on the child´s health. According to the National Family Health Survey 2019-21 in India, children born with a weight of less than 2.5 kg face a higher risk of malnutrition and childhood morbidities, which are leading causes of under-five mortality [10]. Low birth weight children are about 20 times more likely to die than their healthier counterparts and are closely associated with their mortality [13,14]. Similarly, the above study indicated that low birth weight predisposes children to different life problems, including physical, mental growth failure and premature death. Therefore, the evidence from the above sources supports the findings from this study.

**Socio-economic factors of under-five child mortality:** the second objective of this study was to explore socio-economic factors associated with U5M. This study found that the number of household members and partner´s education were the specific factors associated with U5. The study found that the risk of child death was less (97.38% of survived children) among families with many household members, ranging between six to ten (6 to 10 members). Meaning that it was a protective factor. Studies found that a family member, with the mother, father, grandmother, aunt, uncle, or older children, plays an important role in the family to protect a child and form a good shelter for the child [4,15].

This finding was not surprising, and the explanation is that, depending on household structure, there is a role sharing in a family if it is composed of working-age adults and/or grandmothers who are perceived as guardians of young children. The results are not very different from what was found in an SSA study done in 2020, saying that for household members of 3 to 5 and high or equal to 6 children headed by a female, the risk was a 10% significant reduction of under-five mortality [16].

About the partner´s education, the study found that the partner´s level of education was an associated factor with the U5M, mainly for children whose parent has a secondary level, with 97.85% and higher level 98.4% compared to no education. This finding is true based on the results of other studies on the influence of partner schooling on women's health services utilization and frequent antenatal care visits, and skilled birth attendance in developing countries [17].

**Limitation of the study:** findings of this study were only based on data found from RDHS 2019-2020. Meaning that other factors associated with under-five mortality that are not in the stated RDHS records were not fully disclosed. This study encountered some limitations in the literature, where studies done about child mortality were old, not meeting the researcher's needs.

## Conclusion

Under-five mortality was associated mainly with low birth weight and partial compliance with post-natal checkups within two days following labor. While delivery at a health facility was the protective factor, it. A deep analysis should be carried out in the community to find out the reason behind the factors of U5M, especially low birth weight and post-natal care. Furthermore, existing interventions aiming at preventing child mortality should be thoroughly evaluated.

### What is known about this topic

- Mother's education, wealth index, birth defects, infectious diseases, and injuries are major contributors to under-five mortality, particularly in developed countries;
- Bio-demographic factors like the child's sex, mother's age, and birth intervals also impact child mortality, though less frequently documented in studies;
- In sub-Saharan Africa, under-five mortality decreases when mothers complete 3-4 antenatal care visits.

### What this study adds

- Low birth weight is a significant factor associated with under-five mortality;
- Inadequate post-natal check-up within 2 days after childbirth contributes to higher child mortality;
- Household members play a crucial role in protecting against child mortality.
